# Data visiting governance: a conceptual framework

**DOI:** 10.1186/s40246-025-00864-0

**Published:** 2025-12-04

**Authors:** Donrich Thaldar

**Affiliations:** School of Law, University of KwaZulu-Natal, Mazisi Kunene Road, Glenwood, Durban, South Africa

**Keywords:** Genomic data sharing, Data sovereignty, Federate learning, Data localization, Trusted research environments

## Abstract

As genomic research scales globally, legal constraints such as data localization provisions in data privacy and other laws and ethical imperatives around privacy and sovereignty increasingly challenge traditional models of data sharing. Data visiting, where analysis occurs within the provider’s computing environment without moving the data, offers a promising alternative, yet its governance remains underdeveloped. This article introduces the Seven-Dimensional Data Visiting Framework (7D-DVF), a structured tool for designing, assessing, and regulating data visiting systems in genomics. Building on the Global Alliance for Genomics and Health (GA4GH) data sharing lexicon, the framework disaggregates data visiting into seven adjustable dimensions: researcher autonomy, data location, data visibility, nature of the shared data, output governance, trust and control model, and auditability and traceability. Each dimension operates as a governance lever, enabling proportional, context-sensitive configurations that balance privacy, utility, and legal compliance. The article illustrates how the 7D-DVF can guide practical implementation through checklists and real-world scenarios, including institutional data control, Indigenous data sovereignty, and federated AI model training. By shifting genomic governance from reactive compliance to design-based stewardship, the 7D-DVF equips stakeholders to operationalize secure, lawful, and future-ready data sharing practices.

## Background

In an era where genomic datasets are projected to exceed zettabyte scale by 2025—driven by advances in next-generation sequencing and AI-driven analysis—the imperative for secure, equitable data sharing has never been greater. Yet this growth collides with rising regulatory hurdles, including data localization mandates across Africa and Asia that restrict cross-border transfers to protect sovereignty and privacy [1, 2]. *Data visiting*—where data are analyzed within the provider’s controlled environment without being physically moved—offers a promising technical workaround as it can support both compliance with localization provisions in data privacy and other laws and collaboration across borders [3]. Despite its promise, ad hoc implementations of data visiting can lead to governance problems, including variable privacy protections, ambiguous oversight mechanisms, and unequal power dynamics, highlighting the need for a well-considered governance framework.

In an effort to promote clearer communication about emerging data sharing practices, the Global Alliance for Genomics and Health (GA4GH) undertook a consultative process to develop consensus definitions for key terms relating to data visiting [3]. This effort culminated in a lexicon that provides definitional clarity through standardized, plain-language terms intended to promote semantic and technical interoperability in genomic research. At its core is the concept of *data visiting*, defined as a form of data sharing in which data are analyzed within the provider’s computing environment, either by human or computational agents. Related terms include *federated data analysis* (data visiting involving multiple providers), *remote data interrogation* (query-only access without raw data visibility), and *pseudonymized data* (coded data re-identifiable only with a separate key). These definitions reflect GA4GH’s commitment to neutrality and proportionality, offering a useful baseline for governance discussions across diverse research contexts. However, definitional clarity is only a starting point. Practical implementation requires a deeper understanding of data visiting—not just what it is, but how it can vary across different settings. This calls for a structured conceptual framework that disaggregates data visiting into its constituent dimensions to support informed, context-sensitive governance design.

While general data governance frameworks, such as the Five Safes model and the FAIR principles, provide robust guidance for managing data access and stewardship, they are not tailored to the unique challenges of data visiting in genomics. The Five Safes framework, originally developed in 2003 for the UK Office for National Statistics’ Virtual Microdata Laboratory, structures data access decisions around five interdependent dimensions—safe projects (ensuring legitimate purposes), safe people (trusted users with training), safe settings (secure environments like research data centers or remote access systems), safe data (appropriate anonymization levels), and safe outputs (vetting results to prevent disclosure) [4]. Its novelty lies in the multi-dimensional assessment of risks across these integrated controls, emphasizing a holistic, risk-based approach that treats anonymization as a residual rather than primary control and balances research utility with confidentiality in health and social research [4]. This model promotes integrated solutions for data distribution but focuses on general access models rather than the in situ analysis central to data visiting, where data immobility addresses sovereignty and localization provisions. Similarly, the FAIR principles—Findable (e.g., assigning globally unique persistent identifiers and rich metadata), Accessible (e.g., retrievable via standardized protocols with authentication), Interoperable (e.g., using formal knowledge representation languages and qualified references), and Reusable (e.g., clear licensing and provenance)—prioritize machine-actionability to enhance automated discovery, integration, and reuse of scholarly data, including algorithms and workflows, in diverse ecosystems like general-purpose repositories [5]. While FAIR fosters interoperability across fragmented data landscapes and supports computational stakeholders in overcoming barriers to e-Science, it remains domain-independent and high-level, lacking specific levers for genomic contexts involving sensitive pseudonymized data or federated analytics vulnerable to re-identification risks.

The OECD Recommendation on Health Data Governance complements these by outlining 12 high-level principles for national health data frameworks, such as promoting secure data use for public benefit, harmonizing privacy protections, ensuring interoperability, and facilitating transborder cooperation, as implemented across OECD countries from 2016 to 2021 [6]. Although not explicitly focused on data visiting, the OECD framework is technology-neutral but consistent with practices such as federated data analysis and remote data interrogation, aligning with data visiting’s emphasis on minimizing movements to comply with localization requirements, reduce privacy risks, and support evidence-based policy in health crises like COVID-19. Taken together, these frameworks—Five Safes for risk-managed access, FAIR for machine-driven reusability, and OECD for policy harmonization—provide valuable high-level guidance but share a common limitation: they lack operational, configurable tools tailored to the unique governance challenges of data visiting in genomics.

This limitation forms the rationale for the Seven-Dimensional Data Visiting Framework (7D-DVF)—a novel conceptual and operational tool that disaggregates data visiting into seven governance-relevant dimensions. Each dimension can be tuned as a *governance lever* to achieve context-sensitive outcomes, such as enhanced privacy, legal compliance, research utility, or ethical accountability. This flexibility is essential in the face of rising complexity. For instance, genomic research increasingly relies on artificial intelligence, or AI-based, inference and federated analytics, as seen in Parkinson’s disease and Crohn’s prediction studies, where finely tuned system controls are required to manage privacy risks without compromising performance [7, 8]. A unidimensional understanding of data visiting risks oversimplification, particularly in visibility settings where pseudonymized data may still be vulnerable to re-identification [9, 10]. Meanwhile, global inequities persist: in Africa, data governance must simultaneously democratize access and respect localization provisions—pressures that multidimensional design can help reconcile [11, 12]. Furthermore, emerging technologies like homomorphic encryption and FAIR (findability, accessibility, interoperability and reuse) Data Points demand flexible architectures that balance innovation with compliance, extending GA4GH standards into implementable governance tools [13, 14]. Such configurations can serve as governance-by-design levers: technical choices (e.g., encryption, query-only interfaces, provenance services) that implement legal and ethical requirements such as anonymization, purpose limitation, and accountability.

The need for 7D-DVF rests on four core foundations:

1. *Implementation Gaps*: Existing GA4GH lexicon terms are conceptually robust but do not capture practical variation in data visiting systems (e.g., centralized trusted research environments vs. decentralized federated learning), leading to inconsistent privacy, security, and oversight [15].
2. *Regulatory Pressures*: Laws in many jurisdictions impose legal constraints on data transfers, requiring new governance levers to enable collaboration without violating localization mandates [2, 16].
3. *ELSI Advancement*: The framework enables proportional, context-sensitive governance aligned with ethical, legal, and social implications (ELSI). It is particularly relevant in settings such as Indigenous genomics or rare disease research, where stakeholder trust and community engagement are critical [17, 18]. Likewise, data visiting can support institutional claims of data ownership as a strategy to resist exploitative data flows and mitigate data colonialism [19, 20].
4. *Technological Evolution*: As artificial intelligence (AI) and federated learning architectures mature, governance models must accommodate complex trade-offs between trust, auditability, autonomy, and utility [21].

### Thesis

The 7D-DVF unlocks the governance potential of data visiting by providing a configurable framework for designing, assessing, and regulating data visiting practices in genomics. The framework consists of the following seven dimensions.

**Fig. 1:**
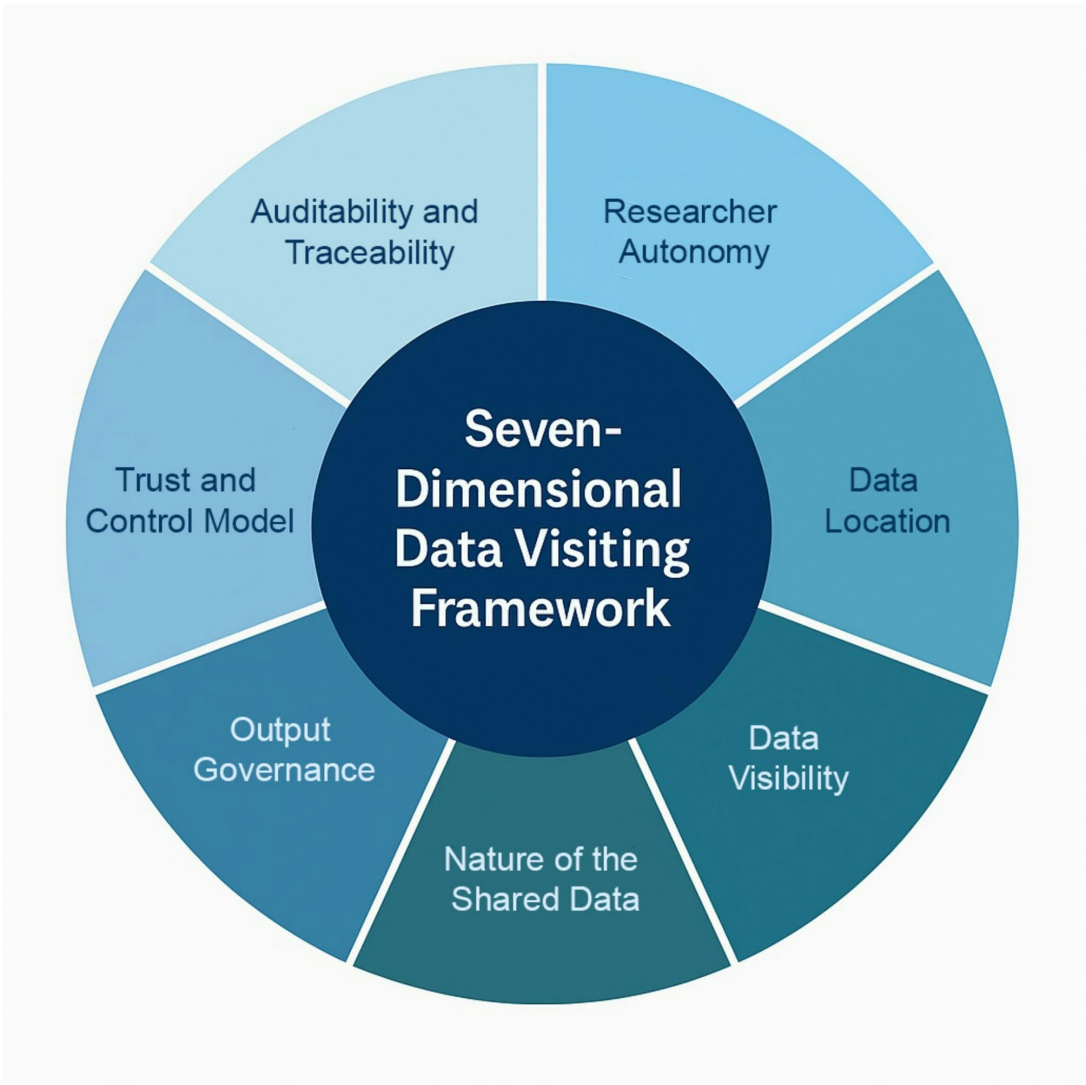
Seven-dimensional data visting framework (7D-DVF). (Source: author)

1. *Researcher Autonomy*: Spectrum from full custom-code execution to fixed queries.
2. *Data Location*: Ranges from centralized cloud hosting to fully decentralized in-jurisdiction analysis.
3. *Data Visibility*: From full access to de-identified datasets to strictly query-based interfaces.
4. *Nature of the Shared Data*: From identifiable to anonymized data types.
5. *Output Governance*: Controls on how analytic results are reviewed, modified, or released.
6. *Trust and Control Model*: Distribution of oversight, ranging from centralized trusted research environments (TREs) to distributed peer control or embedded computational agents.
7. *Auditability and Traceability*: Extent and format of monitoring, from full lifecycle provenance metadata to embedded privacy-preserving auditing (Fig. 1).

## The seven dimensions of data visiting

The 7D-DVF framework was developed through sustained participation in interdisciplinary workshops, technical forums, and policy engagements. During this process, it became apparent that both conceptual and practical discussions of data visiting often suffer from oversimplification—whether in ethical discourse, legal analysis, or computational design. The framework presented here emerged as a response: a structured attempt to unpack the multidimensional nature of data visiting and enable more rigorous, proportional, and context-sensitive governance.

The 7D-DVF disaggregates data visiting into seven governance-relevant dimensions. Each dimension captures a distinct feature of how data visiting systems can be configured, and together they form a flexible matrix for proportional, context-sensitive governance. While analytically separable, these dimensions are often interdependent in practice and can be tuned in combination to balance competing priorities such as privacy, utility, and legal compliance. In what follows, each dimension is treated as a technical lever with governance effect: a design choice that implements legal and ethical requirements.

### Researcher autonomy

Researcher autonomy refers to the degree of freedom granted to users—whether human analysts or computational agents—in interacting with shared data within the provider’s environment. This dimension spans a governance-relevant spectrum:

- High autonomy allows full custom code execution, algorithm development, and exploratory querying;
- Medium autonomy restricts users to pre-approved scripts, tools, or interfaces;
- Low autonomy limits interactions to fixed queries or predefined outputs, often akin to *remote data interrogation*, where users submit requests without direct manipulation [3].

Autonomy captures the balance between analytical flexibility and normative trust in users, directly shaping how data visiting aligns with governance goals such as proportionality, accountability, and compliance. These levels of autonomy are technical system choices, but they also function as governance levers by shaping compliance with legal obligations such as purpose limitation and accountability.

In practice, high-autonomy models are evident in TREs where researchers can run bespoke AI algorithms on genomic datasets, such as variant analysis in the UK Biobank’s secure platform, supporting rapid hypothesis testing but requiring robust safeguards [22]. Medium-autonomy systems are common in federated setups like FedCrohn, where pre-vetted tools facilitate exome-based modelling across institutions without exposing raw data [8]. Low-autonomy configurations dominate privacy-focused platforms—such as rare disease registries or remote *Beacon* queries—where users are confined to standardized interactions to minimize disclosure risks [18, 21, 23].

These implementations illustrate how autonomy modulates research efficiency. High levels can accelerate discovery in multi-omics research, such as federated Parkinson’s studies, but may also expose vulnerabilities if not carefully calibrated [7]. Conversely, low autonomy simplifies compliance with regulations like data localization, but can constrain collaborative and exploratory potential in global consortia [1, 24].

As a *tuneable governance lever*, researcher autonomy enables risk-adjusted design. High autonomy fosters innovation but heightens risks of unauthorized re-identification, calling for compensatory controls such as role-based identity and access management, ethics certification, or dynamic monitoring [25]. In contrast, low-autonomy systems impose stricter constraints ex ante, reducing governance overhead but requiring careful consideration of utility trade-offs.

The dimension is tightly coupled with other axes in the 7D-DVF. High autonomy must be paired with strong output governance (e.g., differential privacy, result vetting), auditability and traceability (e.g., provenance metadata, logging), and context-sensitive data visibility controls. For instance, federated learning tools like COLLAGENE or PPML-Omics allow high-autonomy computation only when output filtering and system-level encryption reduce re-identification risks [26, 27]. In Indigenous genomics contexts, by contrast, community-led restrictions on autonomy may be preferred to preserve trust and collective sovereignty [17]. Configured autonomy is a technical control with governance effect: pre-approved tools and constrained execution operationalize purpose limitation and accountability obligations, while preserving proportionate utility.

Best practices for proportional autonomy management include implementing tiered access controls using identity and access management systems to align user autonomy with their role, training, and jurisdictional context. Where researchers require greater flexibility, custom tools or workflows can be pre-approved by data access committees or ethics boards to ensure compliance without unduly restricting innovation [28]. In high-autonomy environments, autonomy should also be linked to other governance dimensions—for example, by requiring enhanced output controls or real-time audit logging to mitigate risk [25, 26]. Ultimately, the strength of this dimension lies in its configurability: as genomic research becomes more federated and AI-driven, autonomy should not be treated as a binary choice but rather as a governance setting that can be tuned to promote ethical, effective, and legally compliant data visiting.

### Data location

Data location refers to the physical or virtual infrastructure in which shared data reside during visiting. This dimension spans a spectrum: from centralized cloud environments (e.g., provider-controlled platforms compliant with localization laws), to institutional on-premises servers, to distributed systems with unified interfaces (such as national research clouds supporting federated data analysis), and finally to fully decentralized models, where analytic agents operate at the data source without relocating it [3]. Data location is a foundational governance lever—directly shaping who controls data, under which jurisdiction, and subject to which norms of sovereignty, accountability, and trust.

In genomic applications, centralized cloud hosting is common in large-scale projects like the UK Biobank, where provider-managed platforms enable scalable access with internal oversight [22]. Institutional hosting, such as hospital-based rare disease registries, ensures that sensitive health data remain within trusted firewalls and national borders [18]. Distributed models with unified interfaces are seen in multi-country consortia harmonizing population datasets, enabling joint analysis without requiring central storage [23, 28]. Decentralized configurations, using embedded or lightweight agents, are increasingly adopted in African health data initiatives and Indigenous-led genomic projects to uphold sovereignty and avoid extraction [17, 29].

These configurations influence both control and performance. Centralized systems streamline multi-user access and simplify analytics but may increase vendor dependency and cross-border exposure. In contrast, decentralized models enhance institutional control and sovereignty, support equity in low-resource settings, and reduce regulatory friction—yet often demand higher interoperability and may limit certain types of real-time analysis.

From a governance perspective, location is critical for navigating legal frameworks like the General Data Protection Regulation and national localization mandates. Decentralized models can avoid cross-border transfers altogether, limiting jurisdictional risk, but require coordinated metadata standards and shared semantics to prevent balkanization [16]. Centralized models offer efficiency and technical support but necessitate auditing and enforceable agreements to ensure compliance with jurisdiction-specific laws. In sensitive domains like federated multi-omics, location decisions can calibrate sovereignty against collaboration [30].

Neutral best practices for managing data location include conducting jurisdictional audits to identify hosting arrangements that comply with localization provisions and reflect the sensitivity of the data involved [1, 2]. In centralized systems, enforceable service level agreements can help preserve sovereign control and maintain governance alignment without necessitating a complete redesign of existing infrastructure. Where data are hosted in decentralized environments, location governance should be linked to visibility and traceability, for example by restricting data visibility to enhance privacy and reinforce institutional oversight [11, 14]. Closely interdependent with trust models and visibility settings, data location defines the spatial architecture of data visiting, shaping what is possible, permissible, and proportionate in cross-border genomic research.

### Data visibility

Data visibility refers to the extent to which users can view or explore underlying data during a data visit. This dimension spans a spectrum of access configurations. At one end, *full visibility* grants researchers access to datasets. *Partial visibility* restricts access to metadata, previews, or aggregate summaries. This model is commonly employed in federated registries for rare diseases, where participating sites share only high-level information—such as allele frequencies or model parameter summaries—without exposing individual records, thus facilitating cross-site coordination without compromising privacy [18, 21]. The most restrictive form—*restricted visibility* or query-only access—allows users to submit queries to remote datasets without ever seeing the underlying data. Platforms like FAIR Data Points implement this model: queries run locally at each data holder, and only anonymized, aggregated results (e.g., counts or binary responses) are returned [11]. Likewise, the GA4GH *Beacon* protocol enables users to ask if a specific variant exists in a dataset and receive only yes/no or summary-based responses, preserving local oversight [23]. These technical configurations directly implement governance objectives such as anonymization and proportional access.

These visibility configurations directly shape research utility and privacy risk. Full visibility can enhance interpretability and accelerate multi-omics or AI-powered genomic research, especially when complex pattern detection or predictive model development is required. However, it also increases exposure and re-identification risk. In contrast, restricted visibility offers strong privacy guarantees—critical in high-risk or regulation-constrained contexts—but may limit exploratory analysis and result depth [30]. The choice of visibility level must therefore be contextual, calibrated to legal frameworks, data sensitivity, and institutional governance capacity.

From a governance perspective, visibility is a critical lever for managing risk. High-visibility settings demand compensatory safeguards, including consent-based access tiers and automated anonymization or masking protocols [9]. Restricted modes simplify compliance with data localization provisions by limiting the data surface exposed to external users [1, 24]. In Indigenous or equity-focused genomics, community-led governance supports trust, ensuring that data handling aligns with negotiated arrangements [17].

Neutral best practices for managing data visibility include applying consent tiers that scale visibility according to the sensitivity of the data, thereby ensuring ethically proportionate access. Automated tools that provide previews or aggregate-level outputs can help balance data utility with privacy protection [9]. Visibility should also be linked to output governance, for instance by vetting results in restricted environments to prevent indirect data leakage [26, 27]. Interdependent with the nature of the shared data, visibility reinforces privacy safeguards while enabling proportional research access. Its downstream interactions with output governance and auditability make it a pivotal early-stage design choice in data visiting systems.

### Nature of the shared data

The nature of the shared data refers to the type and sensitivity of genomic information shared during visiting. It spans a spectrum from personal data (e.g., clinical records with direct identifiers like names), to pseudonymized data (coded and re-identifiable only with a separate key), de-identified data (identifiers removed but re-identification possible through linkage), and anonymized data (irrevocably stripped of identifiers, rendering re-identification infeasible) [9, 31]. Selecting identifiable, pseudonymized, or anonymized data is a pipeline design decision that maps directly onto legal categories, enabling compliance to be encoded in transformation and access layers. While de-identified data remains within the scope of data protection laws due to residual re-identifiability, anonymized data typically falls outside such frameworks. The situation with pseudonymized data is more complicated, as the law is not always clear and jurisdictions differ in how they regulate or categorize it [32]. This dimension shapes legal obligations, ethical oversight, and technical safeguards, acting as a foundational lever for proportionality in governance.

De-identified data supports broader utility in platforms like the UK Biobank, enabling variant analysis without direct links to individuals [22]. Anonymized data—often in the form of aggregates—facilitates low-risk applications in rare disease registries or Indigenous-led research [17, 18]. These classifications illustrate how the nature of shared data affects the feasibility of collaborative research and the scope of applicable legal obligations.

From a governance perspective, this dimension is critical for regulatory scoping and ethical calibration. Identifiable or pseudonymized data typically triggers stricter oversight—such as ethics committee review and compliance with localization provisions—requiring safeguards to balance utility with re-identification risks [33]. By contrast, anonymized data may lighten oversight burdens, enabling broad global sharing, though it requires technical verification to prevent residual risk [9]. In African contexts, ensuring that data is non-personal may support equitable data sharing without undermining sovereignty [1, 29].

Neutral best practices for managing the nature of shared data include conducting classification audits to align data types with applicable ethical and legal requirements, for example, employing key-secured pseudonymization for sensitive cohorts [33]. Where possible, shifting to anonymized aggregate data can reduce oversight burdens without undermining research objectives [14]. These strategies are often integrated with visibility controls, such as limiting access to query-only views for pseudonymized datasets to support data minimization and mitigate re-identification risks [10, 11]. Closely linked with visibility and output governance, the nature of shared data is central to calibrating privacy risk and ensuring proportionate protections in data visiting systems.

### Output governance

Output governance concerns the controls governing the release and use of results generated through data visiting. It spans a spectrum from unrestricted export of findings, to reviewed outputs (involving manual or automated vetting such as re-identification risk checks or cell size suppression), privacy-enhanced mechanisms (for example, differential privacy via noise injection or result rounding), and pre-approved formats that restrict dissemination to structured, anonymized templates. This dimension governs analysis outputs and serves as a critical lever to prevent downstream risks—such as data leakage—while preserving research value.

In genomic practice, unrestricted output governance is uncommon but may be applied to low-risk anonymized aggregates, such as summary statistics in federated learning for Parkinson’s disease [7]. Reviewed outputs are often in TREs, where manual or automated checks are performed prior to release. Privacy-enhanced mechanisms use methods such as homomorphic encryption or dynamic sampling in genome-wide association studies (GWAS) to reduce inferential risks [13, 34]. Pre-approved formats dominate high-security platforms, mandating rigidly defined output structures in omics federated models [26, 27]. These variations illustrate how output governance shapes utility: enhanced controls protect sensitive inferences in rare disease registries but may delay insights or constrain reproducibility if over-applied [18].

Governance implications position this dimension as central to managing proportionality and residual risk. Unrestricted outputs maximize speed but elevate re-identification vulnerability, necessitating alignment with the nature of the shared data. Privacy-enhanced mechanisms support compliance with localization requirements by obscuring individual-level details, enabling sharing in cross-border settings [1, 35]. In Indigenous or sovereignty-sensitive contexts, governed outputs help foster trust by including benefit-sharing clauses or restricting external dissemination [17].

Neutral best practices for output governance include implementing automated vetting mechanisms, such as differential privacy, to scale safeguards in proportion to data volume [10]. For high-sensitivity outputs, pre-approval workflows aligned with ethics review processes are recommended. Additionally, integrating output controls with traceability systems ensures that any modifications are logged and auditable, strengthening downstream accountability [25]. Closely tied to data visibility and the nature of shared data, output governance fortifies endpoint security and supports trust frameworks that define oversight responsibilities across contexts.

### Trust and control model

Trust and control model refers to how governance is structured. A centralized model relies on a single authority or platform to administer access and monitor compliance. In localized models, oversight is conducted by the institution hosting the data, such as a university or hospital. Distributed models spread responsibility across a network of peers governed by shared standards. Alternatively, some systems embed control mechanisms computationally, relying on secure agents or environments that enforce constraints automatically. This dimension captures how accountability and oversight are distributed [3].

Localized control suits institutional repositories, such as hospital-led rare disease registries, ensuring host-specific compliance [18]. Distributed networks appear in federated consortia, like international genomic databases governed by GA4GH standards, enabling peer-shared trust across borders [15, 23]. Computational models leverage embedded agents, as in privacy-by-design federated learning for omics data, automating controls without human intermediaries [21, 27]. These variations illustrate the model’s impact on collaboration. Distributed approaches enhance equity in underrepresented regions, while centralized ones offer uniform procedures and streamlined oversight [12, 35].

Governance implications make this dimension a vital lever for accountability and proportionality. Centralized models offer clear oversight but risk power imbalances, addressed through data use agreements (DUAs) to ensure ethical distribution. In sovereignty-sensitive contexts, localized models support compliance with national laws while preserving institutional autonomy and data ownership. In both Indigenous genomics and institutional research settings, distributed or computational control fosters supports context-specific governance. However, these models require high interoperability to avoid fragmentation [36].

Neutral best practices for trust and control models include establishing shared DUAs in distributed systems to harmonize governance standards without requiring centralization [15]. In large federated networks, embedding automated agents can provide scalable computational trust and oversight [13]. Localized models benefit from integrating logging mechanisms to enhance transparency and align with auditability goals [11, 29]. Closely linked to auditability and traceability, this dimension distributes the foundations of governance, allowing other controls to reinforce accountability across the entire system.

### Auditability and traceability

Auditability and traceability refer to the capacity to monitor and record user activities and data interactions during visiting, forming a spectrum from full auditability (comprehensive, real-time logs of all actions enabling retrospective review), to limited monitoring (periodic checks or partial logs focused on key events), and embedded traceability (privacy-preserving auditing built into computational workflows, such as automated provenance tracking) [25]. This dimension supports transparency and accountability, functioning as a lever to verify compliance, detect anomalies, and build stakeholder trust in genomic data ecosystems.

In practice, full auditability is implemented in TREs for high-stakes analyzes, such as logging all queries in rare disease registries to enable forensic oversight [18]. Limited traceability is used in federated platforms like those in African health data spaces, where periodic metadata checks balance governance needs with infrastructure constraints [11, 29]. Embedded traceability leverages tools like provenance metadata in secure federated toolkits, automatically capturing data lineage in omics workflows without imposing burdens on users. These modes illustrate the dimension’s value: full auditing enhances accountability in multi-center studies such as GWAS, while embedded approaches support scalability in privacy-sensitive environments. Immutable logs and provenance metadata provide technical evidence for meeting legal accountability obligations, enabling audits, incident response, and regulator-facing verification.

Governance implications position auditability as a vital lever for proportionality and risk mitigation. Full logging ensures accountability in pseudonymized data settings but may raise privacy concerns if not anonymized, requiring integration with ethical review processes. Limited or embedded traceability reduces overhead in low-sensitivity contexts, supporting localization compliance by documenting in situ activities without excessive data generation [12].

Aligned with the GA4GH lexicon, this dimension extends “provenance metadata” (detailing the lifecycle history of data) and “governance” (ethical oversight), enabling traceable federated data analysis without imposing prescriptive monitoring requirements [23, 31]. It complements pseudonymized data by embedding audit trails that minimize re-identification risks while supporting interoperability.

Neutral best practices for auditability and traceability include incorporating provenance metadata to automate trace logging and ensure compliance with minimal manual effort [25]. Audit intensity should be scaled using tools such as anomaly detection, calibrated to the sensitivity of both the data and the analysis. Embedding audits within distributed networks supports mutual accountability and transparent peer oversight, linking this dimension closely to trust models [15]. As the capstone dimension, auditability and traceability reinforce the integrity of data visiting by intersecting with all others, supporting output governance through logged exports, enabling trustworthy oversight, and ensuring proportional safeguards throughout the lifecycle.

### Implications for data governance

The 7D-DVF offers a practical lens for governing data visiting in genomics, transforming abstract GA4GH definitions into actionable configurations that address ethical, legal, and technical challenges. By treating dimensions as interconnected levers, stakeholders—ranging from ethics committees and data access bodies to researchers and regulators—can design, evaluate, and refine systems with proportionality at the core, balancing risks such as re-identification against benefits like accelerated discovery. This section explores how to operationalize the framework, its benefits and challenges, and its broader impacts on global health research. To apply the 7D-DVF, stakeholders can adopt a modular checklist that assesses each dimension against context-specific needs, such as data sensitivity, jurisdictional constraints, and collaborative goals. This tool promotes deliberative governance, ensuring that configurations remain legible and adaptable. For example, in a high-risk scenario such as a research institution seeking to assert ownership and resist extractive data practices, the checklist may recommend on-premise hosting, limited autonomy, strong auditability, and restricted visibility to uphold institutional control. By contrast, for low-risk anonymized aggregates in federated AI training, higher autonomy and partial visibility may optimize utility. Meanwhile, contexts involving community-led research, such as Indigenous or minoritized populations, may call for decentralized hosting and localized trust models to support self-governance and data sovereignty without defaulting to exclusionary or exceptionalist framings [12]. Checklist for Applying the 7D-DVF:

1. *Researcher Autonomy*: Match user freedom to expertise and risk; e.g., restrict queries in sensitive federated models to mitigate misuse [21].
2. *Data Location*: Align data hosting with localization provisions and institutional sovereignty claims; e.g., host data on institutional servers to reinforce ownership and control, or use decentralized storage to avoid cross-border transfers in African consortia [20, 29].
3. *Data Visibility*: Limit exposure to prevent re-identification; e.g., permit query-only access in rare disease registries [8, 11].
4. *Nature of the Shared Data*: Classify sensitivity to calibrate oversight; e.g., anonymize aggregate datasets for reduced ethics review in multi-omics studies [30].
5. *Output Governance*: Vet analytic outputs for privacy risks; e.g., apply differential privacy in GWAS results [13].
6. *Trust and Control Model*: Structure governance appropriately; e.g., assign oversight to the data-hosting institution in localized models to ensure institutional accountability, or embed automated agents for enforcement in federated systems [15].
7. *Auditability and Traceability*: Match monitoring depth to risk level; e.g., use provenance metadata for verifiable logs in TREs [25].

The benefits of this approach are multifaceted. First, it enhances security by enabling calibrated safeguards, such as combining restricted visibility with privacy-enhanced outputs to mitigate leakage risks in functional genomics [9, 26]. Second, it supports legal compliance, for instance by avoiding cross-border data transfers through decentralized location and auditable traceability, aligning with post-Schrems II legal frameworks [16, 24]. Third, it enables diverse assertions of governance, including institutional control in the face of data colonialism and community-led governance mechanisms grounded in trust and local norms [1, 12]. In sum, the 7D-DVF reduces governance assumptions and fosters innovation in AI–genomics integration without ethical trade-offs.

Nonetheless, challenges remain. Interdependencies among dimensions may complicate standardization, for example, high researcher autonomy may require enhanced traceability, increasing computational burdens in low-resource settings [28]. Additionally, contextual variability may lead to over-customization and interoperability gaps unless harmonized using GA4GH standards [23]. The emergence of automation and AI tools introduces new governance risks necessitating sustained empirical and normative inquiry [14, 37]. These challenges can be addressed through pilots in multi-center studies that assess and refine the framework’s practical utility [18, 30].

Broader impacts span policy and implementation. By operationalizing GA4GH terminology, the 7D-DVF informs ethics checklists for data access committees, facilitating proportional reviews in precision medicine. In international collaborations, it bridges sovereignty gaps, not only for communities but also for research institutions seeking to exercise legal rights over their data, thus enabling fair and effective participation in global genomics [20, 29]. Ultimately, the 7D-DVF shifts genomic governance from reactive compliance to proactive, design-led governance, equipping stakeholders to navigate the evolving 2025 data landscape (Table 1).

**Table 1:** Example configurations using the 7D-DVF

Scenario	Key dimensions tuned	Governance outcome
*Institutional Ownership Assertion* (e.g., preventing data exfiltration or unauthorized reuse)	Medium Autonomy, On-Premise or National Hosting, Restricted Visibility, Pseudonymized Data, Reviewed Outputs, Internal Oversight, Full Auditability	Safeguards institutional ownership
*Community Data Sovereignty* (e.g., Indigenous or minoritized group governance)	Low Autonomy, Decentralized Location, Restricted Visibility, Anonymized Data, Enhanced Output Governance, Localized Trust, Full Auditability	Builds trust and supports localized governance
*Federated AI Training* (e.g., model development using low-risk input)	High Autonomy, Centralized Cloud, Partial Visibility, De-identified Data, Privacy-Enhanced Outputs, Distributed Trust, Minimal Auditability	Optimizes scalability with lower oversight burden
*Rare Disease Multi-Center Study* (e.g., secure interoperability across registries)	Medium Autonomy, Distributed Location, Query-Only Visibility, Pseudonymized Data, Privacy-Enhanced Outputs, Computational Trust, Limited Auditability	Enables lawful data visiting at scale

## Conclusion

The 7D-DVF marks a conceptual advance in the governance of data visiting, building on GA4GH’s definitional groundwork to offer a multidimensional framework for context-sensitive system design in genomics [3]. By unpacking data visiting into seven adjustable dimensions, the framework provides a structured approach to calibrating access, oversight, and infrastructure, thereby supporting lawful, proportionate, and inclusive health research across diverse contexts [9, 35].

### Call to action

Stakeholders are encouraged to embed the 7D-DVF into GA4GH-aligned tools—such as lexicons, ethics templates, decision support instruments, and capacity-building resources—and to pilot its use across a spectrum of data sharing configurations. Such field-level applications will help evaluate its feasibility, refine its parameters, and facilitate uptake in policy and practice [23, 33].

### Pandemic preparedness

As demonstrated during the COVID-19 crisis and reiterated in recent European and African policy frameworks [29, 38], rapid, cross-jurisdictional data analysis is critical for early detection, surveillance, and coordinated response. Data visiting, especially when structured through a calibrated framework such as the 7D-DVF, offers a legally and technically feasible model to enable such responsiveness without compromising sovereignty or privacy. Its ability to facilitate in situ analytics, respect local control, and reduce transfer bottlenecks positions it as a foundational element of next-generation pandemic preparedness infrastructure.

### Forward-looking

As federated systems, AI capabilities, and cross-border governance demands evolve beyond 2025, so too must our frameworks. New developments such as synthetic data generation, bias amplification, and multi-agent learning will test the limits of existing safeguards and require adaptive tuning of each dimension. Continued empirical and normative inquiry, especially in underrepresented research settings, will be critical [1, 12]. Rather than a fixed solution, the 7D-DVF positions data visiting as a dynamic, governable infrastructure, that is, one that can support secure, just, and future-ready global genomics.

## Data Availability

Not applicable.

## Abbreviations

7D: DVF Seven-Dimensional Data Visiting Framework
AI: Artificial intelligence
DUAs: Data use agreements
GA4GH: Global Alliance for Genomics and Health
FAIR: Findability, accessibility, interoperability and reuse
ELSI: Ethical, legal and social implications
TREs: Trusted research environments
GWAS: Genome-wide association studies
